# Therapeutic effects of engineered exosome-based miR-25 and miR-181a treatment in spinocerebellar ataxia type 3 mice by silencing *ATXN3*

**DOI:** 10.1186/s10020-023-00695-6

**Published:** 2023-07-12

**Authors:** Zhenchu Tang, Shenglan Hu, Ziwei Wu, Miao He

**Affiliations:** 1 Department of Neurology, The Second Xiangya Hospital, Central South University, 410011 Changsha, Hunan China; 2 Hunan Key Laboratory of Tumor Models and Individualized Medicine, The Second Xiangya Hospital, Central South University, 410011 Changsha, Hunan China

**Keywords:** SCA3, MJD, Exosome, miRNA-25, miRNA-181a

## Abstract

**Background:**

Spinocerebellar ataxia type 3 (SCA3) is the most common autosomal dominant hereditary ataxia worldwide, which is however in a lack of effective treatment. In view of that engineered exosomes are a promising non-invasive gene therapy transporter that can overcome the traditional problem of poor drug delivery, the aim of this study was to evaluate, for the first time, the value of exosome-based microRNA therapy in SCA3 and the therapeutic effects of intravenously administrated *ATXN3* targeting microRNAs in transgenic SCA3 mouse models.

**Methods:**

The rabies virus glycoprotein (RVG) peptide–modified exosomes loaded with miR-25 or miR-181a were peripherally injected to enable targeted delivery of miRNAs to the brain of SCA3 mice. The behaviors, ATXN3 level, purkinje cell and other neuronal loss, and neuroinflammation were evaluated 4 weeks after initial treatment.

**Results:**

The targeted and efficient delivery of miR-25 and miR-181a by modified exosomes substantially inhibited the mutant *ATXN3* expression, reduced neuron apoptosis and induced motor improvements in SCA3 mouse models without increasing the neuroinflammatory response.

**Conclusions:**

Our study confirmed the therapeutic potential of engineered exosome-based miR-25 and miR-181a treatment in substantially reducing ATXN3 aggregation and cytotoxicity by relying on its targeted and efficient drug delivery performance in SCA3 mice. This treatment method shows a promising prospect for future clinical applications in SCA3.

**Supplementary Information:**

The online version contains supplementary material available at 10.1186/s10020-023-00695-6.

## Background

Spinocerebellar ataxia type 3 (SCA3), or Machado-Joseph disease (MJD), is known as the most common autosomal dominant hereditary ataxia, which is caused by abnormal and unstable expansion of a CAG repeat sequence in the *ATXN3* gene. Intranuclear aggregation of the mutant polyglutamine expanded ATXN3 protein is a neuropathological hallmark of SCA3/MJD linking to neurotoxicity, which is associated with neuronal dysfunction and degeneration (Bichelmeier et al. 2007; Matos et al. 2019; Koeppen 2018). It has been shown by previous studies that silencing mutant *ATXN3* in SCA3/MJD animal models is effective in slowing down the disease progression(Rodriguez-Lebron et al. 2013; Nobrega et al. 2013; Nobre et al. 2022). However, there is no cure for SCA3/MJD yet, so finding a safe, effective and non-invasive therapeutic approach is of great value (Chen et al. 2020).

MicroRNAs (miRNAs) are a class of small, non-coding RNAs that regulate the expressions of target genes at the posttranscriptional level (Gebert and MacRae 2019). Recent studies have indicated that miRNAs-mediated regulation plays a significant role in the pathogenesis of SCA3/MJD(Krauss and Evert 2019). The miR-25 and miR-181a, which belong to two representative miRNA families that directly target the 3’ untranslated region (3’ UTR) of *ATXN3* mRNA, were reported to be capable of reducing the mutant ATXN3 level and aggregation* in vitro* (Carmona et al. 2017; Huang et al. 2014). However, *in vivo* studies on their effect in SCA3/MJD transgenic animal models are still in lack.

Establishing a safe, efficient and non-invasive procedure is critical for further clinical applications of nucleic acid-based gene therapies in treating neurological disorders. In this regard, exosomes have been recognized as promising gene therapy nanocarriers in recent years for their potential to protect encapsulated nucleic acids from degradation, ability to penetrate the blood-brain barrier (BBB), as well as low immunogenicity and toxicity (Alvarez-Erviti et al. 2011; Tian et al. 2014; Liang et al. 2020; Kalluri and LeBleu 2020). Rabies virus glycoprotein (RVG) can help target neurons (Izco et al. 2019). After being engineered to the exosomal surface by fusing with protein lysosome-associated membrane glycoprotein 2b (Lamp2b), RVG can help transport exosomal miRNAs specifically into the central nervous system (CNS) (Lai et al. 2020). Peripherally injected RVG-Lamp2b modified exosomes (RVG-Lamp2b Exos) have been proven to be capable of transporting therapeutic nucleic acids to the brain in a safe, rapid and efficient manner for the treatment of several disorders other than SCA3/MJD (Lai et al. 2020; Venkat et al. 2019; Kojima et al. 2018).

**Fig. 1:**
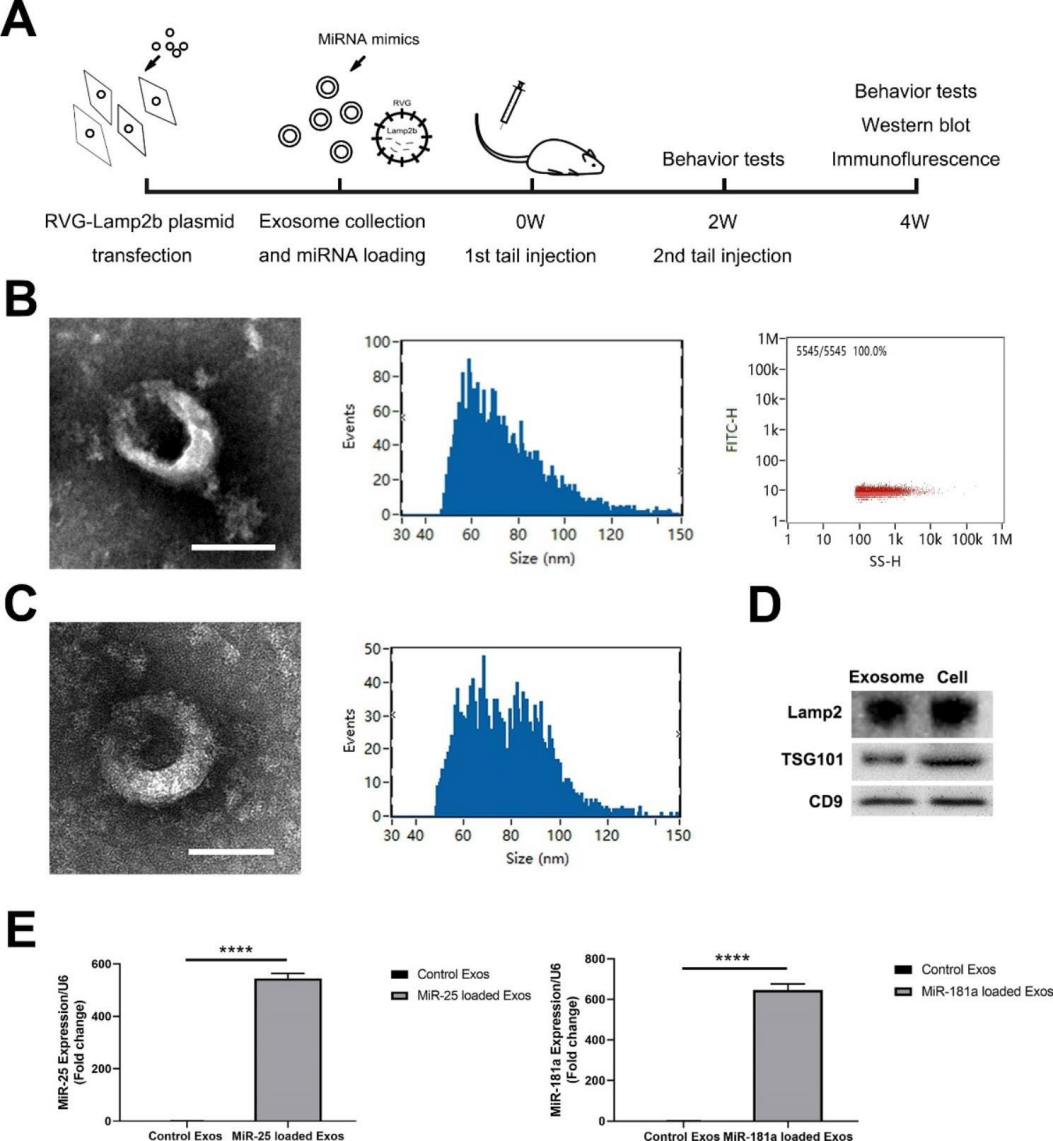
Characterization of engineered exosomes. (**A**) Longitudinal trial design. (**B**) Transmission electron micrograph of RVG-Lamp2b Exos (Scale bar = 100 nm). Size distribution and concentration of RVG-Lamp2b Exos measured by nano-flow cytometry. (**C**) Transmission electron micrograph and size distribution of RVG-Lamp2b Exos after the loading of miR-25. (**D**) Western blotting analysis was performed on the HEK293 cells transfected with RVG-lamp2b and their released exosomes. The protein makers of Lamp2b, TSG101 and CD9 were evaluated. (**E**) The miR-25 and miR-181a were significantly overexpressed in RVG-Lamp2b Exos after miRNA loading, in comparison with unloaded control RVG-Lamp2b Exos. Data were normalized to U6 and presented as fold change relative to the control Exos group. ****<0.0001

In this paper, we tested whether engineered exosomes could achieve targeted delivery of miR-25 and miR-181a to the brain of SCA3 mice and whether miR-25 and miR-181a could alleviate neuropathology by reducing the ATXN3 level *in vivo*. Our investigation is the first of its kind with a particular focus on the exosome-based miRNA therapy for SCA3/MJD. Besides, we also aimed to examine the effects of intravenously administrated *ATXN3* targeting miRNAs (miR-25 and miR-181a) using transgenic SCA3 mouse models for the first time. For the purposes above, we peripherally injected RVG-Lamp2b Exos to enable targeted delivery of encapsulated miRNAs to the brain of SCA3 mice and then evaluated the changes in motor function, ATXN3 level and neuropathological parameters (Fig. 1A).

## Methods

### Cell preparation and transfection

Human Embryonic Kidney (HEK) 293 cells were cultured in Dulbecco’s modified Eagle medium (DMEM) supplemented with 10% fetal bovine serum (FBS) at 37 °C under 5% CO2, and were seeded at 1 × 10^6^ cells per well in a 6-well dish for 24 h. Then pcDNA GNSTM-3-RVG-10-Lamp2b-HA plasmids (Addgene, USA) were introduced into the HEK293 cells using Lipofectamine 3000™ transfection reagent (Thermo Fisher Scientific, USA) according to the manufacturer’s instructions. The medium was replaced with DMEM containing exosome-depleted FBS (SBI, USA) 24 h after transfection, and collected 48 and 72 h after transfection, respectively.

### Isolation of exosomes

Supernatants were centrifuged at 1000 × g for 10 min to remove free cells, at 3000 × g for 20 min to remove cellular debris, and at 10,000 × g for 30 min to remove free organelles in sequence. Then, after ultracentrifugation at 100,000 × g for 70 min at 4 °C, exosome pellets were collected using PBS.

### Transmission electron microscopy (TEM)

Exosome solution was dropped onto a copper mesh and stained with 2% phosphotungstic acid. After the grids were air dried, a TEM (HT-7800, HITACHI, Japan) was used to acquire images at an acceleration voltage of 100 kV.

### Size and concentration analysis of the exosomes

First, 5 µl of exosome solution was taken and diluted to a 30 µl sample in PBS. After testing the standard sample, the exosome sample was then loaded. The size and concentration of exosomes were detected and analyzed using Nano flow cytometry (NanoFCM) (Flow NanoAnalyzer, NanoFCM Inc., China).

### Extraction of miRNAs

The exosome pellets or smashed tissue samples were lysed with TRIzol Reagent (Thermo Fisher Scientific, USA), and the miRNAs were extracted using the miRNeasy Mini Kit (Qiagen, Germany) according to the manufacturer’s instructions. The RNA concentration and purity were then determined by NanoDrop 2000 (Thermo Fisher Scientific, USA).

### Loading of miRNAs

First, 100 µg of exosomes and 5nM of miRNA mimics with or without Cy5 labeling (GenePharma, China) were mixed in 180 µl of Nucleofector™ solution (Cell Line Nucleofector Kit V, Lonza, Germany) and electroporated using a Nucleofector II device (Lonza, Germany). The exosomes were then washed in 4℃ PBS and precipitated again by ultracentrifugation to remove unincorporated miRNA mimics. The efficiency of transfection was validated by RT-qPCR for the purpose of detecting the miRNA levels.

### Real-time reverse transcription quantitative polymerase chain reaction (RT-qPCR)

Reverse transcription was performed using the miRNA First Strand cDNA Synthesis (Tailing Reaction) Kit (B532451; Sangon Biotech Co, Ltd., China) according to the manufacturer’s instructions. Thereafter, RT-PCR analysis was performed using the SYBR miRNA RT-PCR kit (B532461; Sangon Biotech Co, Ltd., China) on a Roche LightCycler 96 instrument (Roche, USA). The PCR program was as follows: 1) 95°C for 30s, 2) 40 cycles at 95°C for 5s and at 60°C for 30s, and 3) melting and cooling at 95°C for 10s, at 65°C for 60s, at 97°C for 1s, and at 37°C for 30s. Each sample was amplified in triplicate. The relative expressions of miRNAs were normalized to U6 expression. All the primers were synthesized by Sangon Biotech Co, Ltd., China. The sequences of the primers were as follows: MiR-25-3p: 5’-TCATTGCACTTGTCTCGGT-CTGA-3’; MiR-181a-5p: 5’-AACATTCAACGCTGTCGGTGAGT-3’.

### Mouse model and genotyping

The SCA3/MJD transgenic mice (B6; CBA-Tg(ATXN3*)84.2Cce/lbezJ) were purchased from The Jackson Laboratory (USA) (Cemal et al. 2002). All procedures regarding the care and use of animals were approved by the Institutional Animal Care and Use Committee of the Second Xiangya Hospital (No. 2021683) and Central South University (No. 2021sydw0016), and all animal testing methods used were strictly in accordance with the institutional regulations and the guidelines of the Hunan Animal Care and Use Committee. After breeding, agarose gel electrophoresis and capillary electrophoresis sequencing were performed for mouse genotyping (Supplemental figure). The expanded CAG repeats were amplified by PCR using the following primers: F: 5’-CCAGTGACTACTTTGATTCG-3’ (with or without FAM labeling); R: 5’-TGGCCTTTCACATGGATGTGAA-3’. The PCR program was as follows: (1) 95 °C for 5 min, (2) 38 cycles at 95 °C for 30s, at 59 °C for 30s and at 72 °C for 30s, and (3) 72 °C for 7 min, then 4 °C forever.

### Injection of exosomal miRNAs

Tail vein injection of exosomes was performed on 12-week-old SCA3 mice. Specifically, 200 µg of RVG-Lamp2b Exos, RVG-Lamp2b Exos/miR-25 or RVG-Lamp2b Exos/miR-181a were tail-injected to 3 groups of 12-week-old SCA3 mice (denoted as the Control-SCA3 group (n = 8), the MiR25-SCA3 group (n = 8) and the MiR181a-SCA3 group (n = 8)) respectively at day 0 and day 14. Besides, a group of wild type littermate mice was set as the Control-Wt group (n = 8).

### Mouse brain immunofluorescence

After fixation in 4% paraformaldehyde and dehydration in sucrose solution, the mouse brains collected were frozen and cut coronally into 20 μm slices, which were then permeabilized with 0.2% Triton X-100 and blocked with 5% fetal bovine serum in PBS for 30 min at room temperature. Subsequently, the brain slices were first incubated with primary antibodies including the anti-Calbindin antibody (c9848, sigma, USA), the anti-Ataxin3 antibody (MAB5360, sigma, USA), the anti-NeuN antibody (ABN78, sigma, USA) and the anti-GFAP antibody (MAB360, sigma, USA) overnight at 4 °C, then washed with PBS for 3 times, and further incubated with secondary antibodies including the Alexa Fluor 594 AffiniPure donkey anti-rabbit IgG (711-585-152, Jackson ImmunoResearch laboratories, USA) and the Alexa Fluor 488 AffiniPure donkey anti-mouse IgG (715-545-150, Jackson ImmunoResearch laboratories, USA) for 2 h at room temperature. Fluorescent images were acquired using a Zeiss LSM 900 Confocal Laser Scanning Microscope (Carl Zeiss, Oberkochen, Germany). The cell quantification and fluorescence intensity analyses were performed by scanning three coronal sections in each group under fluorescence microscope (n = 4) using the ImageJ software (National Institutes of Health, USA). The linear density of purkinje cells was determined as the number of Calbindin-positive purkinje cells/mm. Then, the signal intensity of ATXN3 and GFAP was analyzed, and the number of NeuN positive cells/0.25 mm^2^ in deep cerebellar nuclei was evaluated.

### Western blotting

Cerebellums from different groups were harvested and frozen in liquid nitrogen, and then smashed as appropriate. The samples including cells, exosome pellets or tissues were then lysed with radio-immunoprecipitation assay (RIPA) buffer supplemented with protease inhibitor cocktail and phenylmethanesulfonyl fluoride on ice for 30 min. The proteins were separated by 10% sodium dodecyl sulfate–polyacrylamide gel electrophoresis (SDS-PAGE) and electrophoretically transferred to polyvinylidene difluoride membranes (Millipore, Billerica, MA, USA). After blocking, the membranes were incubated overnight at 4 °C with the primary antibodies including the anti-Lamp2 antibody (ab199946, abcam, UK), the anti-TSG101 antibody (ab125011, abcam, UK), the anti-CD9 antibody (ab92726, abcam, UK) and the anti-Ataxin3 antibody (MAB5360, sigma, USA). Thereafter, the membranes were washed with TBST for three times and were incubated with the diluted secondary antibody for 2 h at room temperature. The protein bands were then visualized using enhanced chemiluminescence (ECL) reagents (Thermo Fisher Scientific, USA) as recommended after washing with TBST for three times, and the films were analyzed using ImageJ.

### Beam-walking test

The beam-walking test was performed on a 100 cm-long wooden beam of 1 cm width once in every 2 weeks at a similar time point. During the test, the beam was suspended at a height of 80 cm above the floor, with the goal end attached to an enclosed box. The mice were trained for 3 days before testing to be able to cross the beam and reach the enclosed box within 60s. Each mouse was given three attempts on the beam, and the average traversal time and number of foot slips were counted for analysis.

### Rotarod test

The rotarod test was performed once in every 2 weeks at a similar time point. Each mouse was trained for 3 days before testing. On the trail day, the mice were tested on a rotarod starting from the speed of 5 rpm, which was then linearly accelerated to 40 rpm. The maximal time for one trial was 5 min, and three parallel trials were conducted on each mouse. The mice were allowed to rest for 15 min between two consecutive trials to prevent exhaustion. The latency to fall off the rotarod was recorded and analyzed.

### Statistical analysis

Statistical analysis was performed using the SPSS Statistics (version 22.0; IBM Corp., USA) or Graphpad Prism (version 9.5.0; Graphpad Software, USA). Data were expressed as means ± SEM unless otherwise specified. Comparisons between two groups were made by Mann-Whitney U test, while multiple comparisons between more than two groups were made by ANOVA, Kruskal-Wallis test or Tukey’s test. Differences were considered significant when p < 0.05.

## Results

### Characterization of engineered exosomes

To prepare RVG-Lamp2b Exos, the HEK 293T cells were transduced with pcDNA GNSTM-3-RVG-10-Lamp2b-HA, and the secreted exosomes from the transfected cells were isolated by sequential ultracentrifugation. The Lamp2 protein levels in the RVG-Lamp2b Exos and parental cells were measured by Western blotting, so as to validate whether the Lamp2 protein had been incorporated into the exosomes (Fig. 1D). As expected, the Lamp2 protein was found to be overexpressed in RVG-Lamp2b Exos, as demonstrated by the abundant Lamp2 bands, compared to the parental cells transfected with RVG-Lamp2b plasmids (Fig. 1D). The morphology of the exosomes was observed using TEM (Fig. 1B), the size and concentration of the exosomes were analyzed using NanoFCM (Fig. 1B). Specifically, the RVG-Lamp2b Exos exhibited typical bilayer membrane structures, with a mean diameter of 74.25 nm and a concentration of 2.53 × 10^10^ particles/ml culture medium. In addition, the exosomal markers TSG101 and CD9 were also highly expressed in RVG-Lamp2b Exos (Fig. 1D). Then, miRNA-25 and miRNA-181a were loaded into RVG-Lamp2b Exos by electroporation, namely RVG-Lamp2b Exos/miR-25 and RVG-Lamp2b Exos/miR-181a, respectively. RT-qPCR revealed that there was a remarkable encapsulation of miR-25 or miR-181a into RVG-Lamp2b Exos (p < 0.0001) (Fig. 1E). The mean diameter of exosomes increased slightly to 77.81 nm after the loading of miR-25 (Fig. 1C).

### RVG-Lamp2b Exos can effectively transport miR-25 and miR-181a to the brain

**Fig. 2:**
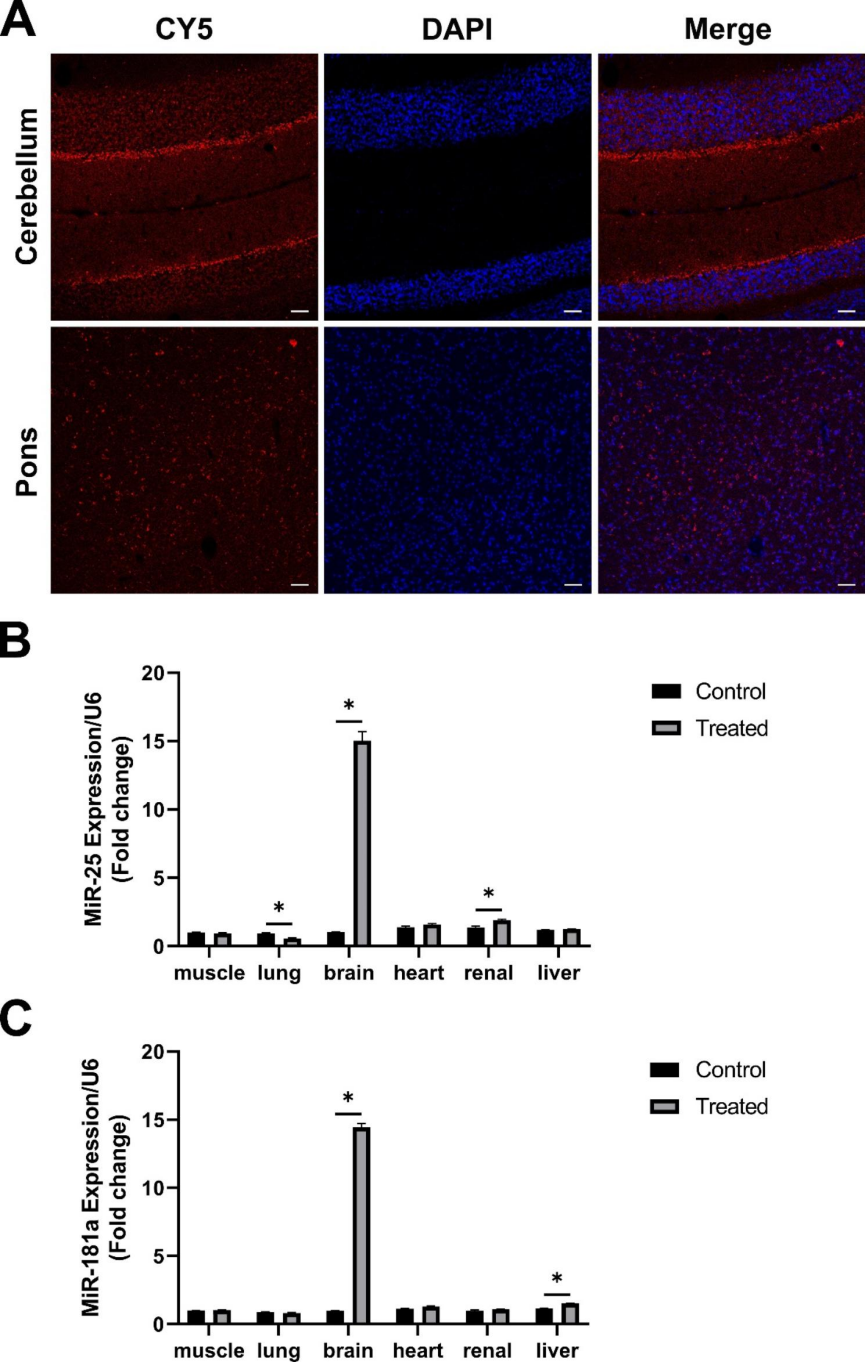
RVG-Lamp2b Exos effectively delivered miRNAs into the brain. (**A**) *In vivo* tracking of Cy5-labelled miR-25 in the cerebellum and pons of SCA3 mouse brain 6 h after tail-vein injection of RVG-Lamp2b Exos/miR-25. Scale bar = 50 μm. (**B**) Comparison of the miR-25 levels in different tissues between control and intravenous RVG-Lamp2b Exos/miR-25 treated group measured by qRT-PCR. (**C**) Comparison of the miR-181a levels in different tissues between control and intravenous RVG-Lamp2b Exos/miR-181a treated group measured by qRT-PCR. Data were normalized to U6 and presented as fold change relative to the control muscle group. *<0.05

In order to analyze the potential targeting ability of the RVG-Lamp2b Exos loaded with miRNAs *in vivo*, 200 µg of RVG-Lamp2b Exos loaded with Cy5 labelled miRNAs were tail-injected to the SCA3 mice. The immunofluorescence results obtained at 6 h after the injection showed that signals from Cy5 labelled miRNAs were detected in the brain, including the regions of cerebellum and pons (Fig. 2A and Supplemental figure). Besides, major organ tissues were collected at 6 h after injection, and the qRT-PCR results further supported that the levels of miR-25 and miR-181a were remarkably increased in the brain compared to untreated control and other tissues (including muscle, lung, heart, renal, and liver, Fig. 2B C). Specifically, the levels of miR-25 or miR-181a in the brain after treatment were over 14-fold higher than those of untreated control. The off-target effects on other tissues were small (Fig. 2B C). The combined evidence confirmed the capacity of RVG-Lamp2b modified exosomes for the targeted and efficient delivery of encapsulated miRNAs to the mouse brain.

### Exosomal miR-25 and miR-181a can improve the motor ability and balance of SCA3 mice

To explore the effect of RVG-Lamp2b Exos/miR-25 and RVG-Lamp2b Exos/miR-181a, 200 µg of RVG-Lamp2b Exos, RVG-Lamp2b Exos/miR-25 or RVG-Lamp2b Exos/miR-181 were tail-injected to 3 groups of 12-week-old SCA3 mice (denoted as the Control-SCA3 group (n = 8), the MiR25-SCA3 group (n = 8) and the MiR181a-SCA3 group (n = 8)) respectively at day 0 and day 14. Besides, a group of wild type littermate mice was set as the Control-Wt group (n = 8).

**Fig. 3:**
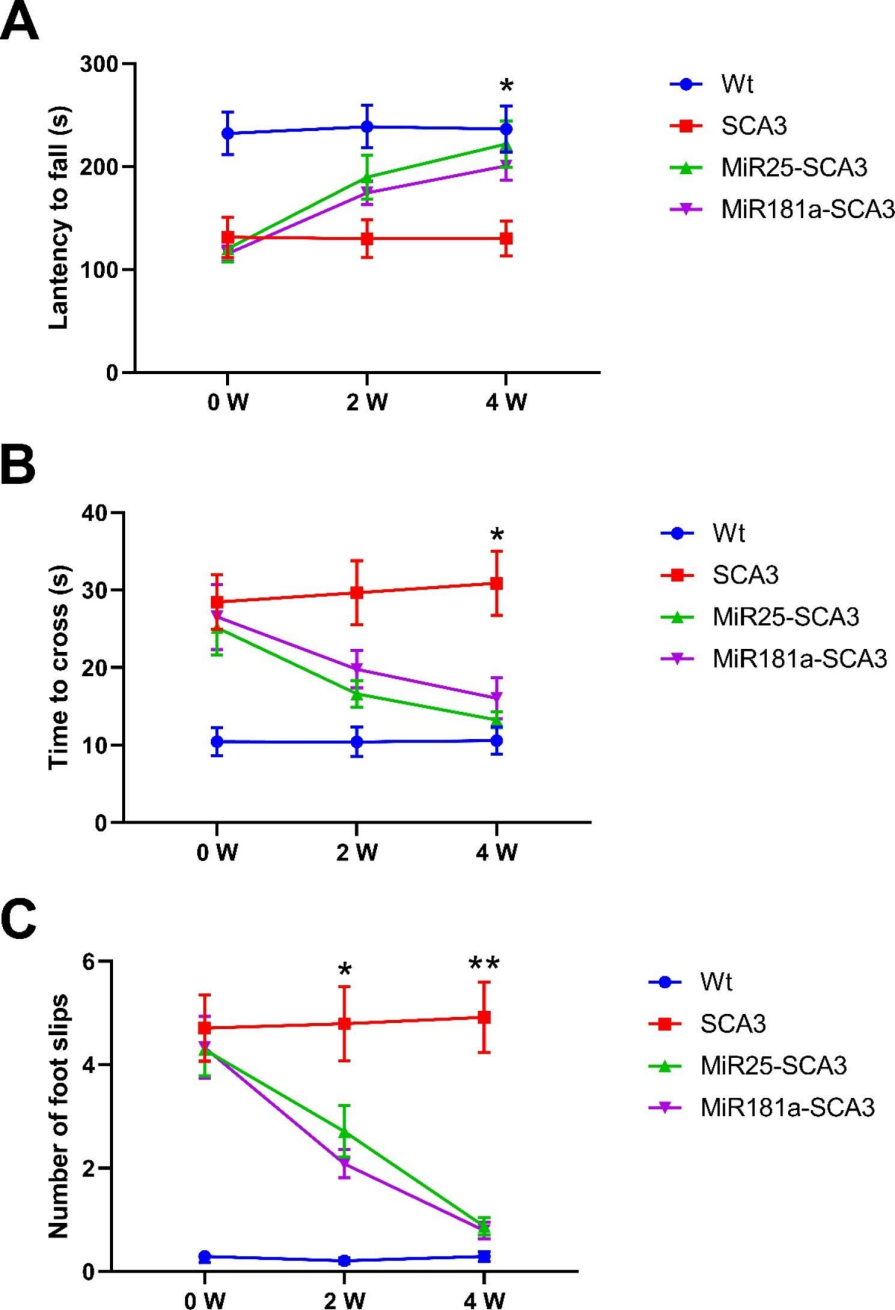
Exosomal miR-25 and miR-181a significantly improved the motor function. (**A**) Rotarod test was conducted 2 weeks and 4 weeks after initial tail-vein injection of exosomal miRNAs. The latency to fall time was analyzed. (**B**, **C**) Beaming-walking test was conducted 2 weeks and 4 weeks after initial exosomal miRNA treatment. The crossing time and number of foot slips were analyzed. N = 8 in each group. Significant differences were detected between the two exosomal miRNA treated groups and the Control-SCA3 group in the rotarod test and beam-walking test at week 4 (at week 2 and 4 for the foot slip numbers). *<0.05, **<0.01

Then, rotarod test and beam-walking test were conducted to evaluate whether the targeted exosomal miRNA treatment could alleviate motor deficit *in vivo*. As shown in Fig. 3A, the Control-SCA3 group exhibited a significantly shorter latency to fall time compared to the two exosomal miRNA treated SCA3 groups, while no difference was found between the three groups before the treatment. At the time point 4 weeks after initial treatment, the average latency to fall time was 221.79 ± 22.56s for the MiR25-SCA3 group, and 200.63 ± 13.73s for the MiR181a-SCA3 group, which were both closer to that of the Control-Wt group (236.5 ± 22.31s) and significantly higher than that of the Control-SCA3 group (130.13 ± 16.76s).

For the beam-walking test, both exosomal miRNA treated SCA3 mice groups exhibited significantly reduced crossing time and foot slips compared to the Control-SCA3 group (crossing time 30.88 ± 4.13s, foot slip number 4.92 ± 0.68) (Fig. 3B C). The crossing times for the MiR25-SCA3 group and the MiR181a-SCA3 group were 13.25 ± 1.05s and 16.04 ± 2.67s respectively, with the average foot slip numbers of 0.88 ± 0.17 and 0.79 ± 0.17, which were comparable to that of the Control-Wt group (crossing time 10.58 ± 1.78s, foot slip number 0.29 ± 0.10). As can be seen, there were significant differences between the two exosomal miRNA treated groups and the Control-SCA3 group in both the rotarod test and beam-walking test at week 4 (p < 0.05) (Fig. 3). The results above indicated that the targeted exosomal miR-25 and miR-181a treatment successfully improved the balance and motor coordinative impairment in SCA3. Though the MiR25-SCA3 group presented a slightly superior performance in the rotarod test and beam-walking test compared to the MiR181a-SCA3 group, the differences were not statistically significant.

### Exosomal miR-25 and miR-181a can attenuate the apoptosis of purkinje and deep cerebellar nuclei cells

**Fig. 4:**
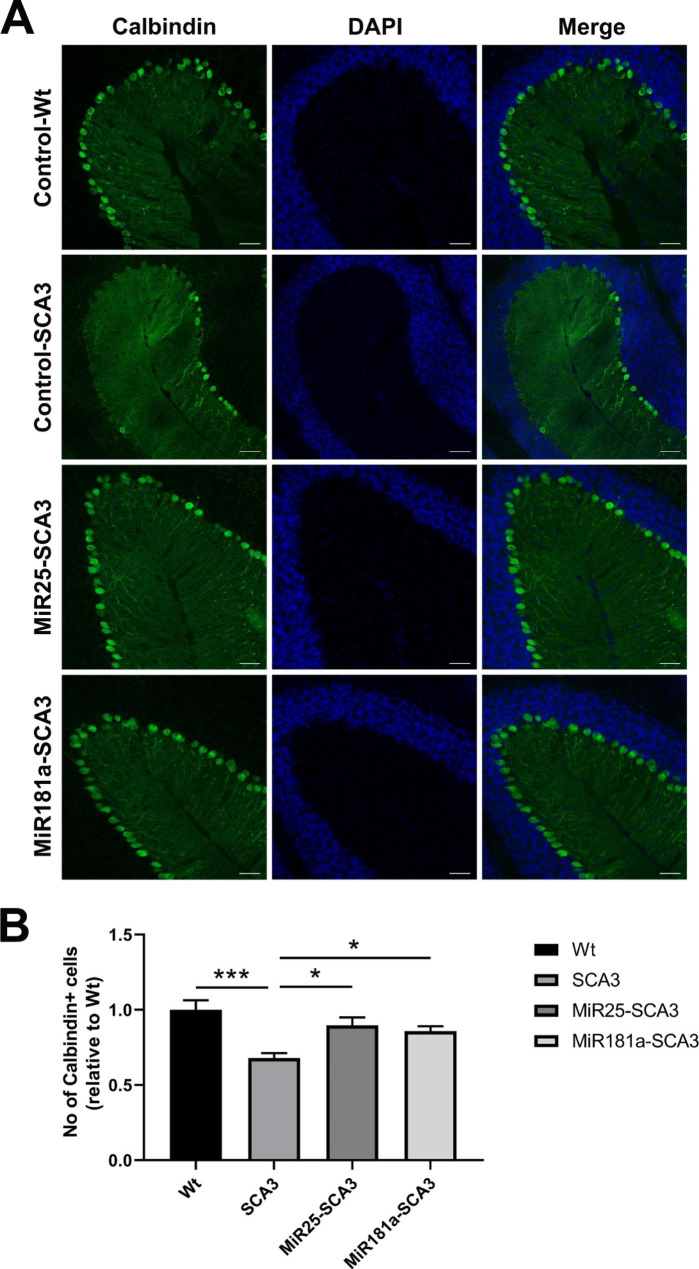
Exosomal miRNA treatment reduced the apoptosis of Calbindin positive cells. (**A**, **B**) Histological and quantitative analysis of Calbindin positive cells in the sagittal cerebellar Sect. 4 weeks after initial injection. Scale bar = 50 μm. *<0.05, ***<0.001

**Fig. 5:**
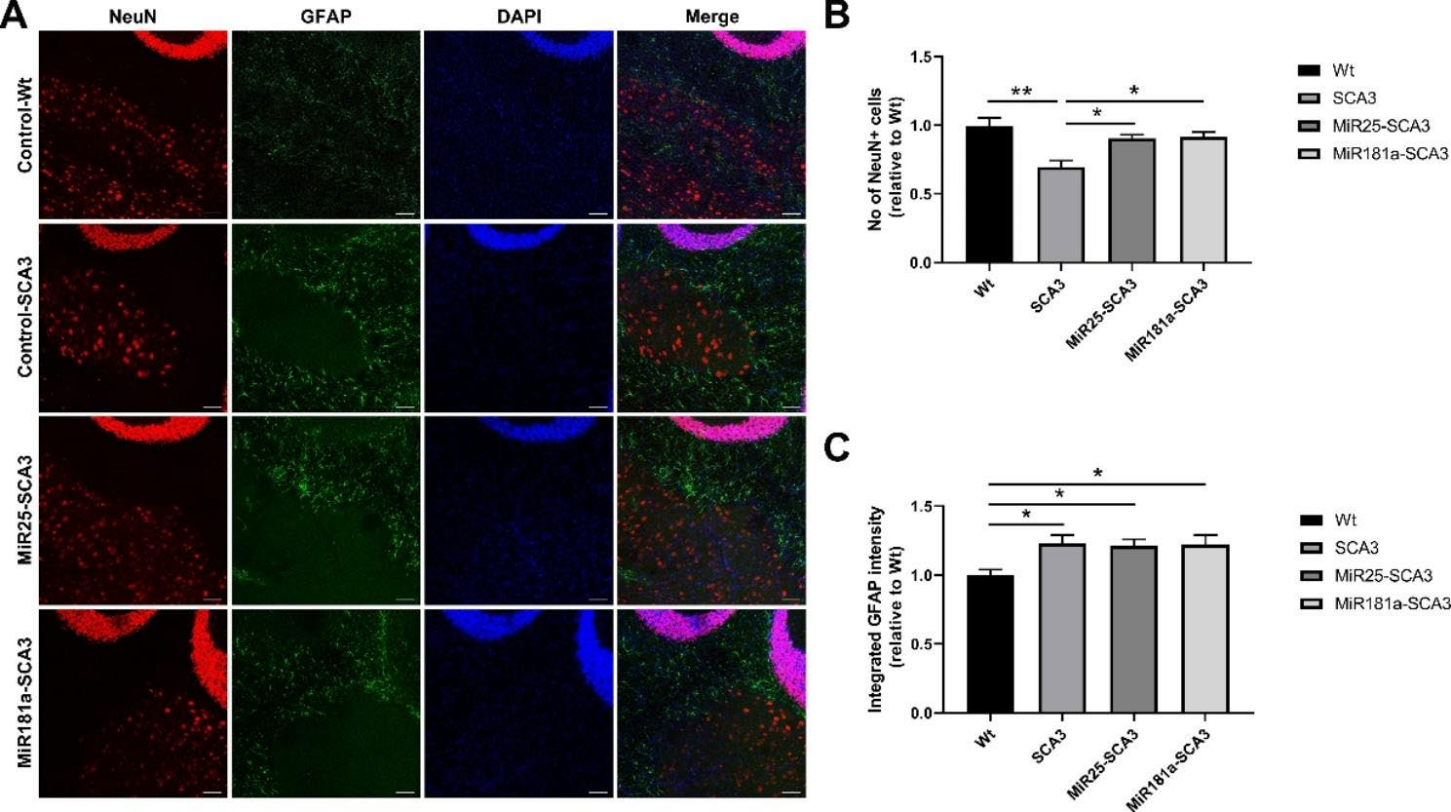
Exosomal miRNA treatment reduced the neuron apoptosis in the DCN without increasing the neuro-inflammatory response. (**A**, **B**) Histological and quantitative analysis of NeuN positive cells in the DCN 4 weeks after initial injection. (**A**, **C**) Immunofluorescent staining and quantitative analysis of the fluorescence intensity of GFAP 4 weeks after initial injection. Scale bar = 50 μm. *<0.05, **<0.01

The apoptosis of purkinje cells and deep cerebellar nuclei (DCN) neurons was evaluated 4 weeks after initial exosomal miRNA treatment by immunofluorescence (Figs. 4 and 5). It was found that both exosomal miR-25 and miR-181a treatment significantly decreased the neuronal loss in the purkinje cell layer (p < 0.05) and DCN (p < 0.05). The numbers of calbidin-positive purkinje cells in the MiR25-SCA3 and MiR181a-SCA3 groups were 1.32 ± 0.18 fold (p < 0.05) and 1.26 ± 0.11 fold (p < 0.05) higher than that in the control SCA3 mice, respectively (Fig. 4). Besides, the numbers of NeuN-positive cells in the DCN of the MiR25-SCA3 and MiR181a-SCA3 groups were 1.3 ± 0.09 fold (p < 0.05) and 1.32 ± 0.12 fold (p < 0.05) higher than that in the control SCA3 mice (Fig. 5). Although the numbers of purkinje cells and DCN neurons in exosomal miRNA treated mice were slightly lower than those in the Control-Wt group, the differences were not statistically significant.

Then, the GFAP level was compared by immunofluorescence to address the possibility of reactive gliosis response to the exosomal miRNA treatment. As a result, no significant difference was detected in the GFAP signal intensity level between exosomal miRNA treated SCA3 mice and control SCA3 mice, though the Control-Wt group showed a lower GFAP staining level (p < 0.05) (Fig. 5).

### Exosomal miR-25 and miR-181a can suppress the mutant ATXN3 level in SCA3 mice

**Fig. 6:**
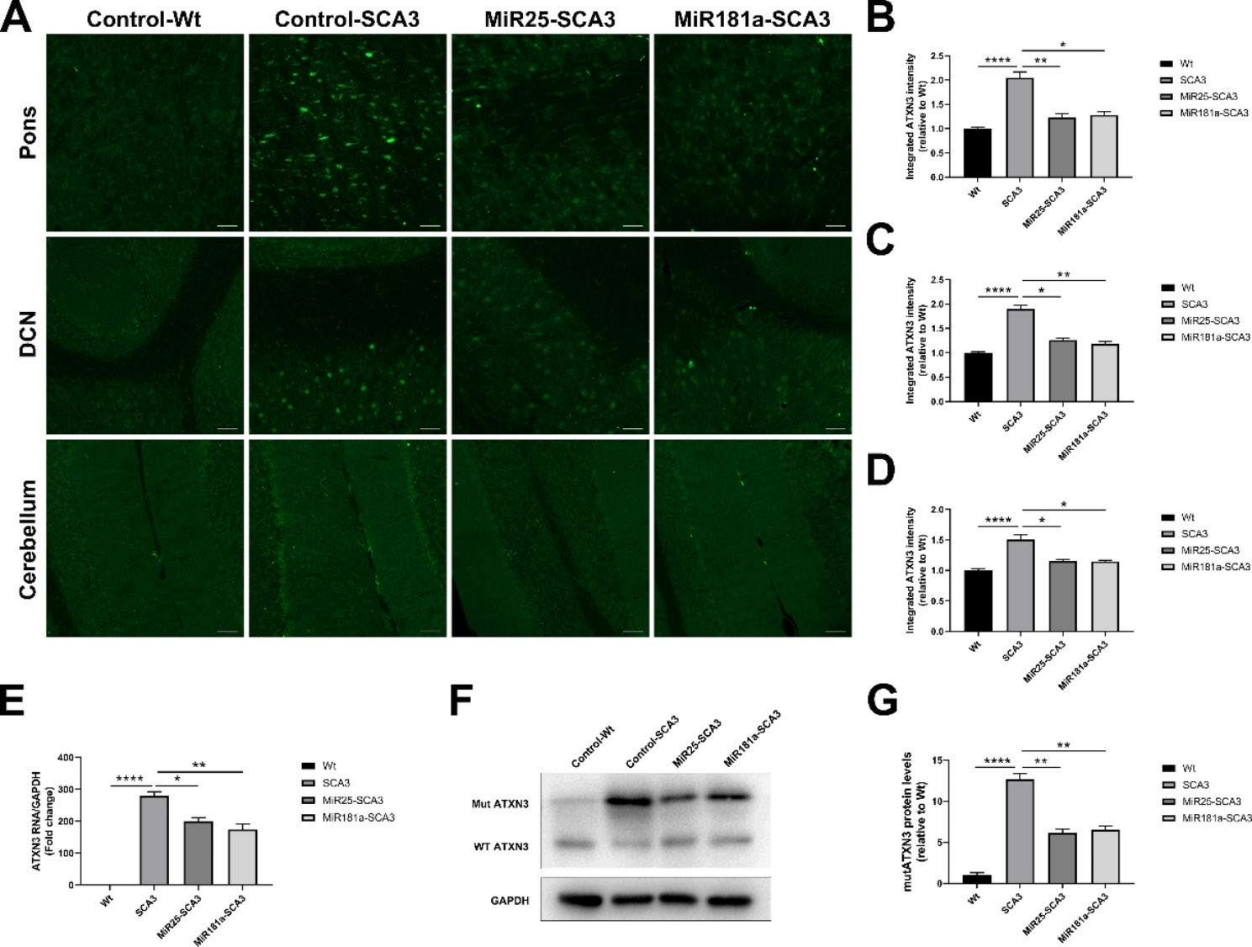
Effect of exosomal miRNAs on the ATXN3 level. (**A**-**D**) Exosomal miR-25 and miR-181a treatment significantly reduced the mutant ATXN3 level in the pons, DCN and cerebellum as evidenced by the quantification of fluorescence intensity 4 weeks after initial injection. Scale bar = 50 μm. (**E**) Analysis of the cerebellar *ATXN3* mRNA level in different groups normalized to GAPDH. (**F**-**G**) The cerebellar mutant ATXN3 protein level in different groups as confirmed by Western blotting. *<0.05, **<0.01, ****<0.0001

The targeted delivery of miR-25 and miR-181a was found to result in a robust reduction in the ATXN3 level in the pons, DCN and cerebellum (especially purkinje cells), primarily in nuclei (Fig. 6). As revealed by quantification of the fluorescence intensity, both exosomal miR-25 and miR-181a significantly reduced the ATXN3 signal in the pons, DCN, and cerebellum (p < 0.05) (Fig. 6A-D). The cerebellar *ATXN3* mRNA level was markedly lowered in both the MiR25-SCA3 (reduced by 28.53%±5.31%, p < 0.05) and MiR181a-SCA3 (reduced by 37.69%±5.12%, p < 0.01) groups compared to the Control-SCA3 group (Fig. 6E). Moreover, Western blotting analysis of whole cerebellar lysates also indicated varying degrees of mutant human ATXN3 protein suppression following the exosomal miR-25 (reduced by 50.55%±4.89%, p < 0.01) and miR-181a (reduced by 47.5%±4.64%, p < 0.01) treatment, which were in agreement with the immunofluorescence and mRNA data (Fig. 6F and G).

## Discussion

In the present study, we recognized a potential therapeutic value of engineered exosome-based miRNA treatment in SCA3/MJD mouse models. It was revealed that the targeted delivery of miR-25 and miR-181a by modified exosomes significantly inhibited the mutant *ATXN3* expression, reduced neuron apoptosis and induced motor improvement in SCA3 mouse models without increasing inflammatory response. To our best knowledge, this is the first study that reported a promising therapeutic effect of SCA3/MJD treatment by delivering miRNAs via engineered exosomes and the first that evaluated the* in vivo* effect of miR-25 in SCA3/MJD.

Establishing a safe, efficient and targeted delivery system is crucial for the successful clinical application of miRNA-based therapeutics (Lu et al. 2018). Compared to other nanoparticles, exosomes are of great nanodrug delivery value for their high physicochemical stability and biocompatibility, low toxicity and immunogenicity, and ability to penetrate the BBB( Venkat et al. 2019; Jahangard et al. 2020). Besides, exosomes are more accessible and safer than artificially synthesized liposomes as drug carriers, since they are endogenous and have already played a critical transporting role in the human body (Alvarez-Erviti et al. 2011; Kalluri and LeBleu 2020; Conceicao et al. 2016). RVG is a neuron-targeting short peptide of the rabies virus glycoprotein, while Lamp2b is a surface binding protein abundantly expressed in exosomes, which can help transfer RVG to the exosomes after co-transfection. Upon being modified by RVG, the exosomes can enable noninvasive and efficient transfer of encapsulated miRNAs to the target neurons with reduced toxicity to other non-targeting cells (Lai et al. 2020; Venkat et al. 2019). The miRNAs delivered by RVG-Lamp2b Exos are able to treat all disease-relevant regions in the brain, rather than a circumscribed effect around the injection site (Lai et al. 2020; Venkat et al. 2019; Kojima et al. 2018). In our study, we confirmed the encapsulation of miR-25 and miR-181a in RVG-Lamp2b Exos. The size of the exosomes was found to increase slightly after miRNA encapsulation, which is possibly attributed to the loading procedure. Our data further confirmed that the peripherally-injected exosomal miR-25 and miR-181a successfully reached the affected regions in the SCA3 mouse brain and provided behavioral and neuropathological recovery without increasing the neuro-inflammatory toxicity. Besides, the off-target effects on other tissues did exist but were small, which we believe can be further reduced by technological improvements in miRNA encapsulation and exosome isolation.

Unlike plasmid DNAs and viral vectors, miRNAs pose no risk of insertional mutagenesis that is resulted from genomic integration (Yin et al. 2014). Both miR-25 and miR-181a were found to be dysregulated in different tissues of SCA3/MJD patients (Carmona et al. 2017; Shi et al. 2014). Previous investigations have indicated that miR-25 and miR-181a could suppress *ATXN3* expression by directly binding to the 3’UTR of *ATXN3* (Carmona et al. 2017; Huang et al. 2014). However, *in vivo* studies that investigate their effect in the spread brain regions of SCA3 animal models are still in lack. It was reported that miR-25 could suppress the aggregation of mutant ATXN3 protein *in vitro* and alleviate cell apoptosis in a SCA3/MJD cell model (Huang et al. 2014). Unfortunately, no further attempts of *in vivo* application in SCA3/MJD animal models have been made. The stereotaxic co-injection of miR-181a and lentiviral vectors encoding for mutant *ATXN3*-3’ UTR in the striatum of C57/BL6 mice was claimed to be able to reduce the mutant ATXN3 level and aggregation, but this study was limited by the circumscribed affected brain region, invasive experimental approach and lack of behavioral test (Carmona et al. 2017). As indicated by our study, the SCA3 mice treated by exosomal miR-25 and miR-181a exhibited a remarkably reduced ATXN3 level in the pons, DCN and cerebellum, as well as attenuated neuron loss in the purkinje cell layer and DCN, which led to subsequential alleviation of motor impairment and inhibition of SCA3 progression. Such results were consistent with previous *in vitro* studies (Carmona et al. 2017; Huang et al. 2014). According to the current evidence, the weakening of cytotoxic effect mediated by mutant ATXN3 aggregation can be associated with decreased neuronal apoptosis and dysfunction, which is also in agreement with previous studies (Nobrega et al. 2013). In addition, our results showed that miR-25 and miR-181a at the same doses could induce similar reductions of the mutant ATXN3 level and neuroprotective effect, though slight differences were observed. Moreover, only two peripheral injections of 200 ug miRNA-loaded exosomes were administrated in our study, suggesting a high delivery efficiency and long-lasting therapeutic effect of the treatment. The combined evidence above supported the potential of engineered exosome-based miR-25 and miR-181a therapy as a promising non-invasive and efficient therapeutic strategy for SCA3/MJD.

Though it was reported that the adeno-associated viruses (AAV)-based miRNA gene therapy could also knock-down the *ATXN3* gene expression, the virus vectors used in such therapy might induce unwanted host immune responses and be integrated into the host chromosome (Ronzitti et al. 2020). Besides, previous *in vivo* studies were mainly based on the method of invasive injection (Nobre et al. 2022; Ronzitti et al. 2020; Martier et al. 2019). Instead, in this study, we selected exosomes that are endogenous with lower immunogenicity as delivery vehicles, and adopted a non-invasive therapeutic approach. A limitation of our study is that the follow-up of the treatment was relatively short (4 weeks), so whether the beneficial effects would be observed in a longer term remains uncertain. Based on the current results, we speculate that exosomal miR-25 or miR-181a administrated intravenously every other week would result in long-term silencing of the *ATXN3* expression. To test this hypothesis, a longer-term analysis should be the focus of future research.

## Conclusion

The results of our study confirmed the therapeutic potential of engineered exosome-based miRNA therapy for SCA3. The brain-targeted delivery of miR-25 and miR181a by exosomes was found to be able to improve the neurological pathology and motor deficits in SCA3 mouse models by inhibiting the mutant *ATXN3* expression. The strategy proposed in this preclinical study, as a non-invasive and efficient treatment, has shown a promising prospect for future clinical application.

## Electronic supplementary material

Below is the link to the electronic supplementary material.

Supplementary Material 1

Supplementary Material 2

## Data Availability

The data that support the findings of this study are available in the body of the text and supplemental materials.

## Abbreviations

SCA3: Spinocerebellar ataxia type 3
RVG: rabies virus glycoprotein
MJD: Machado-Joseph disease
MicroRNAs: miRNAs
3’ UTR: 3’ untranslated region
BBB: blood-brain barrier
Lamp2b: lysosome-associated membrane glycoprotein 2b
CNS: central nervous system
RVG-Lamp2b Exos: RVG-Lamp2b modified exosomes
HEK: Human Embryonic Kidney
DMEM: Dulbecco’s modified Eagle medium
TEM: Transmission electron microscopy
RT-qPCR: Real-Time reverse transcription quantitative polymerase chain reaction
RIPA: radio-immunoprecipitation assay
SDS-PAGE: sodium dodecyl sulfate–polyacrylamide gel electrophoresis
ECL: enhanced chemiluminescence
DCN: deep cerebellar nucleus
