# Supplementary material for: Therapeutic effects of engineered exosome-based miR-25 and miR-181a treatment in spinocerebellar ataxia type 3 mice by silencing ATXN3
Source: Mol Med. 2023 Jul 12;29:96. doi: 10.1186/s10020-023-00695-6 (PMC10337053; doi:10.1186/s10020-023-00695-6)
Supplement: Supplementary file 1 — Supplementary Material 1 [file 10020_2023_695_MOESM1_ESM.docx]

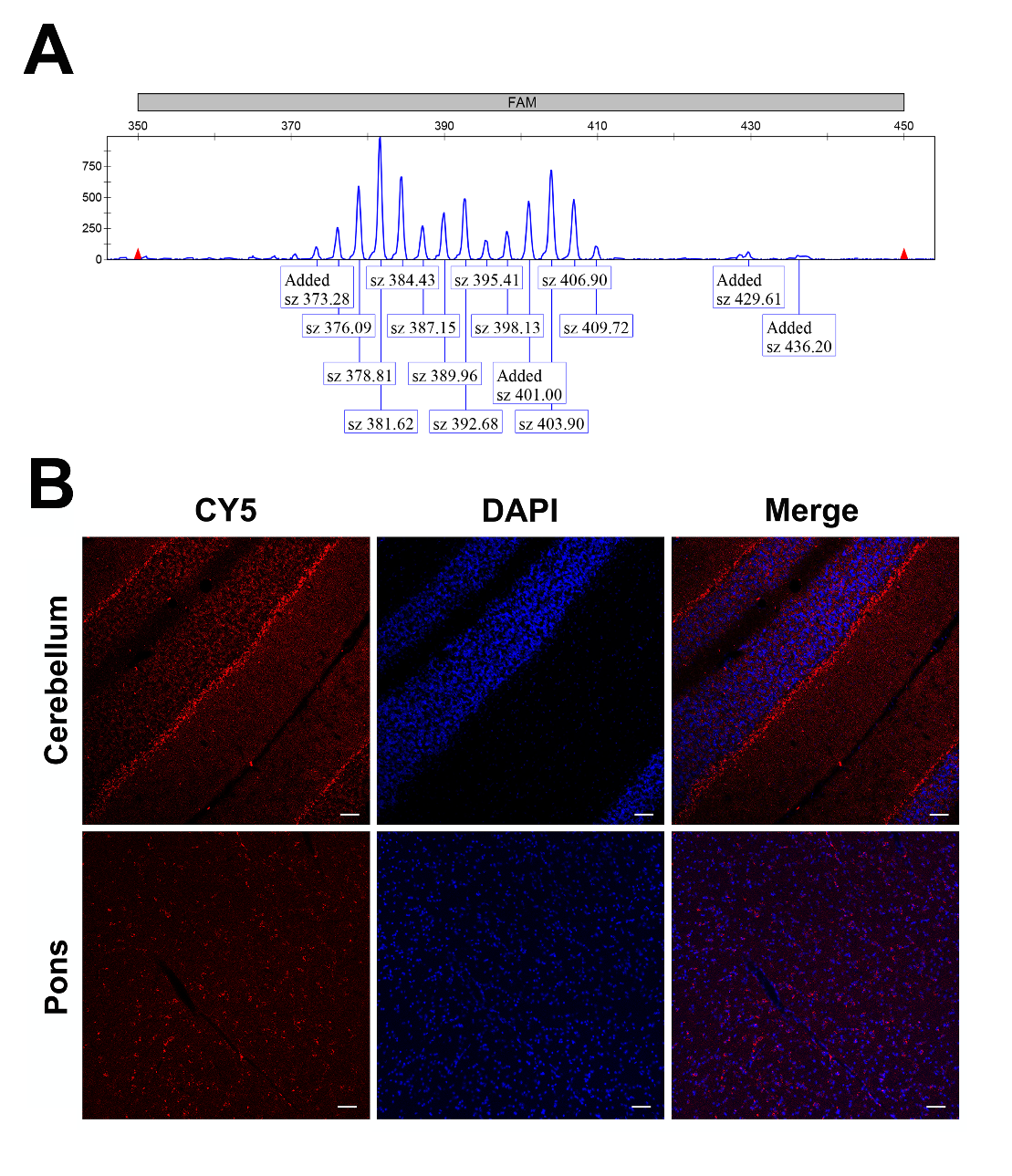


Supplemental figure. (A) Capillary electrophoresis sequencing was performed for mouse genotyping; (B) In vivo tracking of Cy5-labelled miR-181a in the cerebellum and pons of SCA3 mouse brain 6h after tail-vein injection of RVG-Lamp2b Exos/miR-181a. Scale bar=50µm.
