# Supplementary figures and images for: Therapeutic effects of engineered exosome-based miR-25 and miR-181a treatment in spinocerebellar ataxia type 3 mice by silencing ATXN3
Source: Mol Med. 2023 Jul 12;29:96. doi: 10.1186/s10020-023-00695-6 (PMC10337053; doi:10.1186/s10020-023-00695-6)

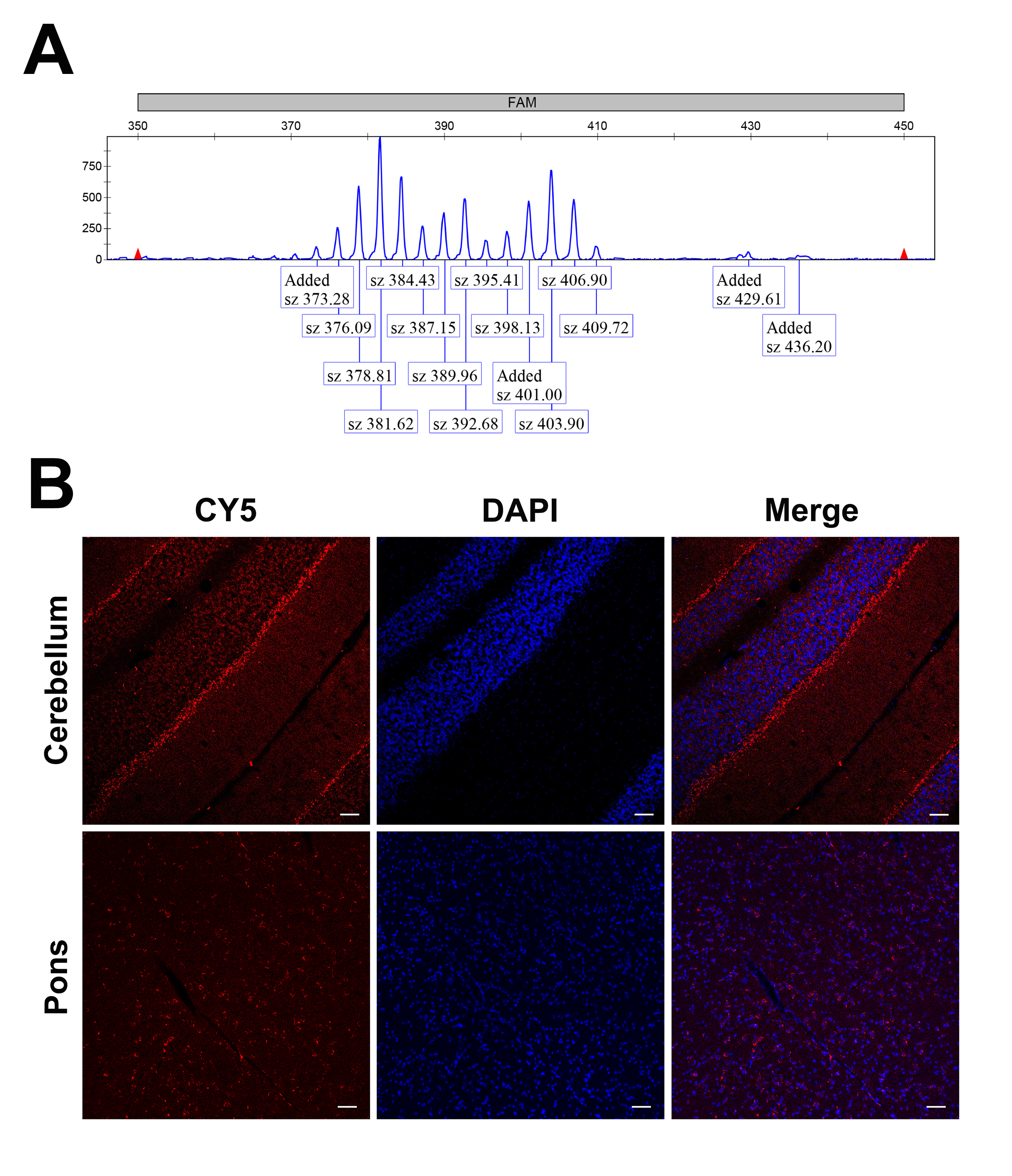

Supplement: Supplementary file 2 — Supplementary Material 2 [file 10020_2023_695_MOESM2_ESM.jpg]
